# SIRT2 Affects Primary Cilia Formation by Regulating mTOR Signaling in Retinal Pigmented Epithelial Cells

**DOI:** 10.3390/ijms21062240

**Published:** 2020-03-24

**Authors:** Jeaho Lim, Juyoung Son, Jaewook Ryu, Ja-Eun Kim

**Affiliations:** 1 Department of Biomedical Science, Graduate School, Kyung Hee University, Seoul 02447, Korea; 338449@khu.ac.kr (J.L.); jysbio@khu.ac.kr (J.S.); busterray@khu.ac.kr (J.R.); 2 Department of Pharmacology, School of Medicine, Kyung Hee University, Seoul 02447, Korea

**Keywords:** SIRT2, mTOR, cilia, cell cycle

## Abstract

SIRT2, a member of the Class III HDAC family, participates in diverse cellular processes and regulates several pathological conditions. Although a few reports show that SIRT2 regulates the cell cycle, the causes and outcomes of SIRT2-dependent cell proliferation remain unclear. Here, we examined the effects of SIRT2 suppression in human RPE1 cells using siRNA targeting SIRT2, and AK-1, a SIRT2-specific inhibitor. The number of primary cilia in SIRT2-suppressed cells increased under serum-present conditions. Suppressing SIRT2 induced cell cycle arrest at G0/G1 phase by inactivating mammalian target of rapamycin (mTOR) signaling, possibly through mTORC1. Treatment with torin 1, an inhibitor of mTORC1/mTORC2, yielded results similar to those observed after SIRT2 suppression. However, SIRT2 suppression did not affect primary cilia formation or mTOR signaling following serum starvation. This suggests that SIRT2 acts as a critical sensor that links growth factor-dependent signal transduction and primary cilia formation by regulating the cell cycle.

## 1. Introduction

SIRT2 is a sirtuin family protein with NAD^+^-dependent lysine deacetylase activity [1], although a recent study shows that SIRT2 also mediates demyristoylation [2]. SIRT2 regulates various physiological and pathological processes, including glucose and lipid metabolism, chromatin remodeling, cytoskeletal organization, genome integrity, motility, autophagy, inflammation, oxidative stress responses, neurodegeneration, and tumorigenesis [3,4]. In addition, SIRT2 governs cell cycle progression, specifically at mitosis, replication phase, and G1/S transition. SIRT2 interacts and deacetylates Cdc20 and CDH1, both coactivators of APC/C (anaphase-promoting complex/cyclosome), and promotes normal progression from mitosis to G1 [5]. SIRT2 also regulates mitotic arrest following exposure to oxidative stress or microtubule depolymerizers [6,7,8,9]. SIRT2-mediated deacetylation of CDK9 or ATRIP is required for recovery from replicational stress [10,11]. Previously, we showed that inhibiting SIRT2 activity using AK-1, a specific inhibitor, causes colon carcinoma cells to arrest at G1, possibly due to inactivation of the NF-κB/CSN2/Snail pathway [12]. Thus, SIRT2 is a critical factor that governs cell cycle, and thereby the balance between cell proliferation and quiescence.

Cilia, which are ubiquitous in nearly all types of human cell, are elongated from the distal end of the basal body (mother centriole) and protrude from the cell surface into the extracellular space [13]. They are microtubule-based structures comprising nine outer doublet microtubules inter-linked along the axoneme, either with or without two central single microtubules [13,14]. The former (9+2), in which the outer microtubules are linked by radial spokes and dynein arms, are motile cilia, whereas the latter (9+0; which lack the radial spokes and dynein arms) are non-motile; non-motile cilia are referred to as primary cilia. Motile and primary cilia are responsible for motility and sensory function, respectively. Cilia are large multiprotein complexes comprising >650 proteins [15,16]. Among these, the intraflagellar transport (IFT) complex plays a major role in bidirectional movement of ciliary proteins and signaling molecules along axonemal microtubules [17]. The tubulin within outer double microtubules undergoes post-translational modifications, including acetylation and glutamylation, which affect cilia assembly and disassembly [18,19].

Primary cilia sense mechanical and chemical extracellular signals [20]; indeed, they transmit signals mediated by Hedgehog, Wnt, Notch, Hippo, G-protein coupled receptors, transforming growth factor-β (TGF-β), insulin-like growth factor 1 (IGF1), and platelet-derived growth factor receptor α (PDGFRα), all of which regulate diverse cellular processes [21,22]. Primary cilia are linked to the cell cycle [23,24,25,26]. Signaling via primary cilia coordinates the cell cycle; however, the cell cycle also affects the timing of primary cilia growth. Primary cilia are present in a quiescent or differentiated state, but they are then resorbed upon re-entry to the cell cycle. In proliferating cells, primary cilia start to be assembled in G1 and are disassembled during S/G2 [27]. 

Previously, we reported that AK-1 treatment results in cell cycle arrest at G1 [12]. Recently, Zhou et al. reported that depletion of SIRT2 increases the number of cells with primary cilia and the length of these primary cilia in mouse IMCD3 (inner medullary collecting duct-3) cells [28]. Therefore, we hypothesized that SIRT2 regulates ciliogenesis via the cell cycle. Here, we show that suppressing SIRT2 inhibits cell proliferation and promotes primary cilia formation under nutrient-present conditions, suggesting that SIRT2 forms a link between energy status and cell proliferation.

## 2. Results

### 2.1. Suppression of SIRT2 Induces Primary Cilia Formation

**Figure 1:**
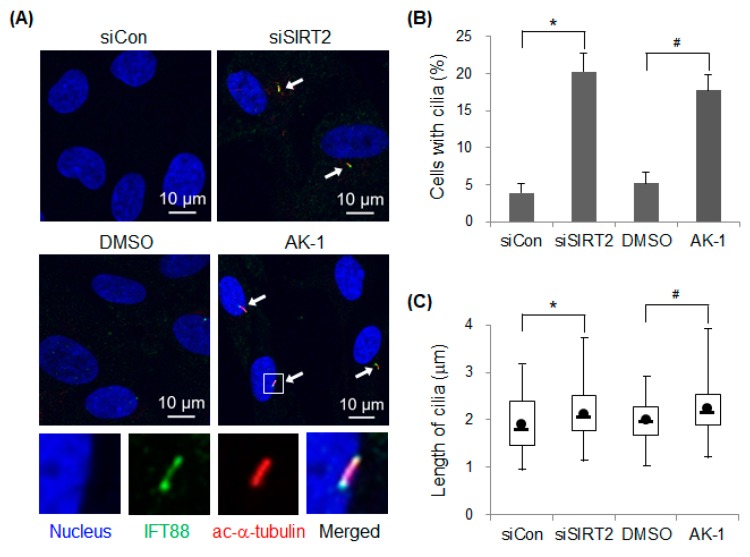
Suppression of SIRT2 increases ciliogenesis in the presence of serum. (**A**,**B**) hTERT-RPE1 cells were transfected with control siRNA (siCon) or SIRT2-targeting siRNA (siSIRT2), or treated with 0.1% DMSO or 10 μM AK-1, a SIRT2-specific inhibitor, for 48 h. (**A**) Cilia were visualized by staining with antibodies specific for IFT88 (green) and α-tubulin-acetyl K40 (red), and nuclei were stained with Hoechst. Arrows indicate cilia and cilia within rectangle are enlarged below. Scale bars, 10 μm; (**B**) The number of ciliated cells (out of 200 cells) was counted. The result is expressed as the mean ± standard error of the mean (SEM; *n* = 6); (**C**) The length of the cilia was measured and is represented in a box-and-whisker plot. A total of 60 cilia were observed from six-independent experiments. Boxes represent interquartile range; whiskers, minimum and maximum values; circles, average values; line, median values. * *p* < 0.05 and # *p* < 0.05; significantly different from siCon-transfected and DMSO-treated cells, respectively (unpaired Student’s *t*-test).

To determine whether SIRT2 affects primary cilia formation, expression or activity of SIRT2 was suppressed in hTERT-RPE1 cells; this cell line is used commonly to investigate cilia assembly and disassembly because it shows robust cilia growth and uniform cilia length [29,30]. Expression and activity of SIRT2 were suppressed by transfecting cells with a specific SIRT2-targeting siRNA or by treatment with AK-1, a SIRT2-specific inhibitor, respectively. Cilia were visualized by staining with anti-IFT88 and acetylated α-tubulin antibodies (Figure 1A). IFT88 is a component of IFT complex B, which is linked to a kinesin-2 motor and is required for anterograde transport from the ciliary base toward the ciliary tip [17,31]. Acetylation of Lys 40 on α-tubulin, catalyzed by α-tubulin acetyl-transferase, is a post-translational modification observed in ciliary microtubules [32]. In the presence of serum, the number of cilia-containing control siRNA-transfected cells was low; however, the number was significantly higher in cells transfected with SIRT2 siRNA. In addition, AK-1-treated cells had significantly more cilia than DMSO-treated cells, suggesting that the catalytic activity of SIRT2 is critical for regulating cilia formation (Figure 1B). Next, we asked whether SIRT2 participates in cilia assembly or disassembly. The length of cilia significantly increased in SIRT2-suppressed cells (Figure 1C), indicating that SIRT2 plays a negative role in cilia assembly in hTERT-RPE1 cells. Overall, the data suggest that both expression and activity of SIRT2 controls cilia formation.

### 2.2. Suppression of SIRT2 Induces a Non-Proliferating Status

**Figure 2:**
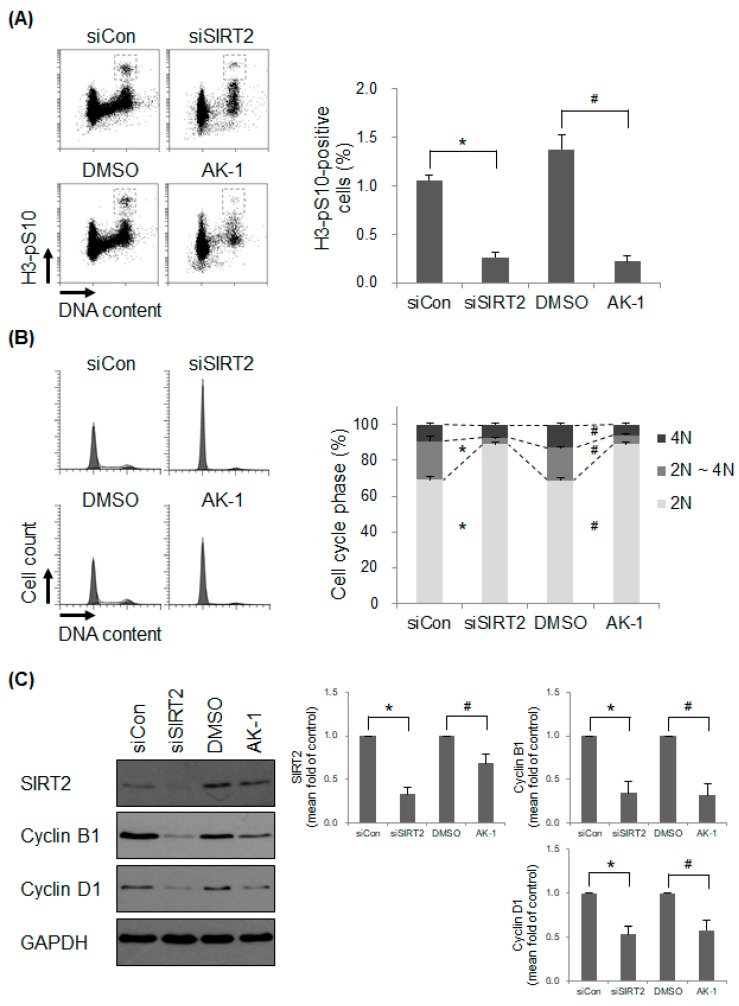
Suppression of SIRT2 induces cell cycle arrest at G0/G1 phase. (**A**–**C**) hTERT-RPE1 cells were transfected with control siRNA (siCon) or SIRT2-targeting siRNA (siSIRT2), or treated with 0.1% DMSO or 10 μM AK-1 for 48 h. (**A**,**B**) Mitotic cells and DNA content were determined by flow cytometry after staining cells with an anti-histone H3-pS10 antibody and propidium iodide. The proportion of mitotic cells (**A**) and the percentage of cells at each phase of the cell cycle (**B**) are expressed as the mean ± SEM (*n* = 5). * *p* < 0.05 and # *p* < 0.05; significantly different from siCon-transfected and DMSO-treated cells, respectively (unpaired Student’s *t*-test). (**C**) Expression of each protein was evaluated by Western blotting. Relative expression is presented as the mean ± SEM (*n* = 4). * *p* < 0.05 and # *p* < 0.05; significantly different from siCon-transfected and DMSO-treated cells, respectively (Mann–Whitney *U*-test).

Next, we asked whether SIRT2 affects cell proliferation, which results in primary cilia formation. Proliferation status was confirmed by examining the cell cycle profile. We found that expression of H3-pS10, a mitosis marker, in SIRT2 siRNA-transfected cells and AK-1-treated hTERT-RPE1 cells was significantly lower than that in corresponding control cells (Figure 2A). The data indicate that SIRT2-suppressed cells do not respond to mitogenic signals under serum-present conditions. In addition, the 2N DNA-containing population within SIRT2-suppressed cells increased, suggesting cell cycle arrest at G0/G1 phase (Figure 2B). Next, we examined expression of SIRT2 and cell cycle-specific cyclins (cyclin B1 and cyclin D1) in SIRT2-suppressed cells. The level of SIRT2 was downregulated markedly upon transfection of SIRT2 siRNA. However, unexpectedly, expression of SIRT2 also decreased following AK-1 treatment, although the reason remains unclear (Figure 2C). As expected, expression of cyclin B1, the levels of which rise specifically during late G2 and M phase, decreased in SIRT2-suppressed cells (Figure 2C). These data are consistent with those presented in Figure 2A. In addition, expression of cyclin D1, the levels of which rises in early G1 phase, decreased in SIRT2-suppressed cells (Figure 2C). This suggests that cells are arrested in a non-proliferating state. Taken together, the data suggest that SIRT2 suppression inhibits cell proliferation, thereby promoting formation of primary cilia.

### 2.3. Suppression of SIRT2 Attenuates mTOR Signaling

**Figure 3:**
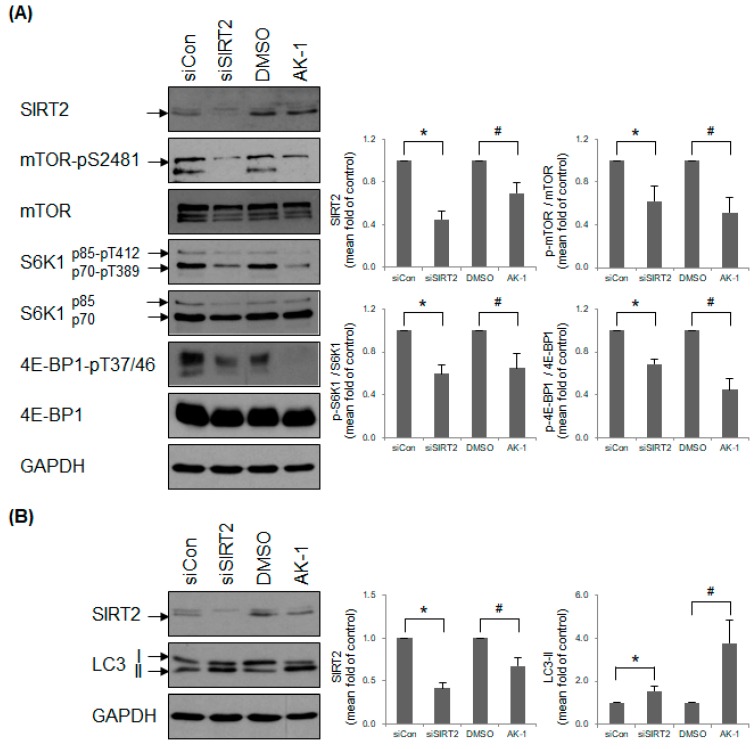
Suppression of SIRT2 inhibits mammalian target of rapamycin (mTOR) signaling. (**A**,**B**) hTERT-RPE1 cells were transfected with control siRNA (siCon) or SIRT2-targeting siRNA (siSIRT2), or treated with 0.1% DMSO or 10 μM AK-1 in the presence of serum for 48 h. Expression of mTOR, its substrates, and LC3 was determined by Western blotting. Relative expression is presented as the mean ± SEM (*n* = 6). * *p* < 0.05 and # *p* < 0.05; significantly different from siCon-transfected and DMSO-treated cells, respectively (Mann–Whitney *U*-test).

We found that SIRT2 affects the number of cilia-containing cells by forming a link between the cell cycle and proliferation (Figure 1 and Figure 2). Therefore, we hypothesized that the effects of SIRT2 on primary cilia formation might be dependent on mitogen- and nutrient-dependent signaling pathways. Mammalian target of rapamycin (mTOR) governs cell growth, proliferation, and survival by sensing mitogens and nutrients [33,34,35]. mTOR kinase functions as multiprotein complexes, mTOR complex 1 (mTORC1) and 2 (mTORC2), both of which contain distinct subunits [36]. Inhibiting mTOR induces formation of primary cilia [37]. This prompted us to investigate whether mTOR signaling in hTERT-RPE1 cells is affected by SIRT2 suppression. We found that both SIRT2 siRNA-transfected cells and AK-1-treated cells expressed low levels of mTOR-pS2481, an autophosphorylated residue and a marker of mTOR catalytic activity [38,39] (Figure 3A). The substrates for active mTORC1 include two isoforms (p70 and p85) of ribosomal protein S6 kinase 1 (S6K1) [40,41] and eukaryotic initiation factor 4E binding protein 1 (4E-BP1) [42,43], both of which are integral for mRNA translation [43,44]. SIRT2 suppression reduced mTORC1-mediated phosphorylation of p70S6K1-T389/p85S6K1-T412 and 4E-BP1-T37/46 (Figure 3A). This is consistent with the finding that suppressing SIRT2 results in low expression of cyclin D1 protein (Figure 2C), one of the target proteins regulated by 4E-BP1-dependent translation [45]. In addition, mTORC1 inhibits autophagy by phosphorylating and inactivating ULK1 [46]. The effect of SIRT2 on mTORC1-mediated autophagy inhibition was determined by measuring the level of LC3-II (the phosphatidylethanolamine conjugate form) [47,48]. The amount of LC3-II in SIRT2-suppressed cells was greater than that in control cells, indicating that SIRT2 is a negative regulator of autophagy induction (Figure 3B). To rule out the possibility of SIRT2 affecting autophagy completion, SIRT2-suppressed cells were treated with chloroquine or bafilomycin A1, which completely blocks autophagosome clearance. The amount of LC3-II was still higher in SIRT2-suppressed cells (Figure S1), indicating that SIRT2 regulates an early stage of autophagy. Overall, these data demonstrate that SIRT2 is required for activation of mTOR signaling.

### 2.4. Suppression of SIRT2 Induces Cilia Formation and Inhibits mTOR Signaling in a Serum-Dependent Manner

**Figure 4:**
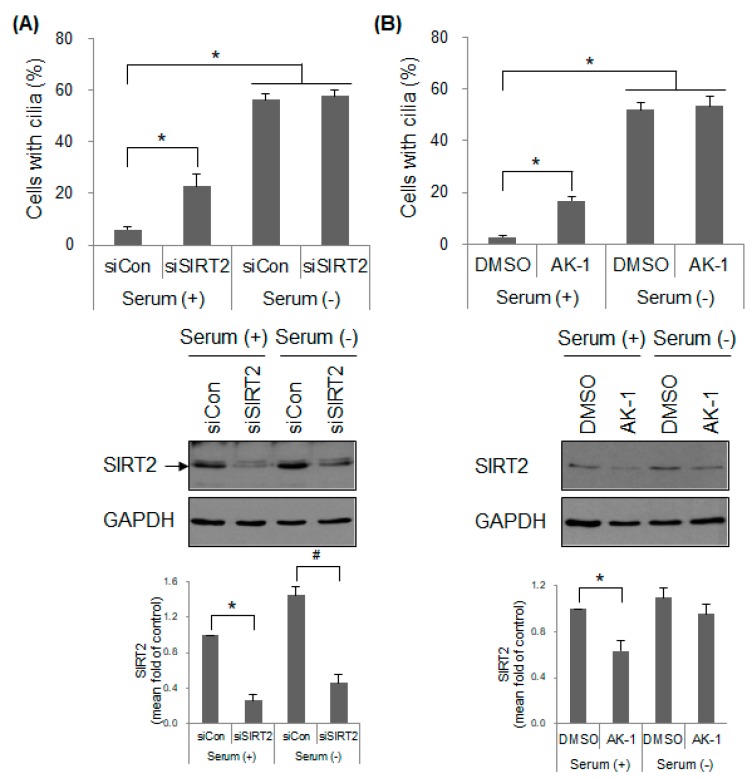

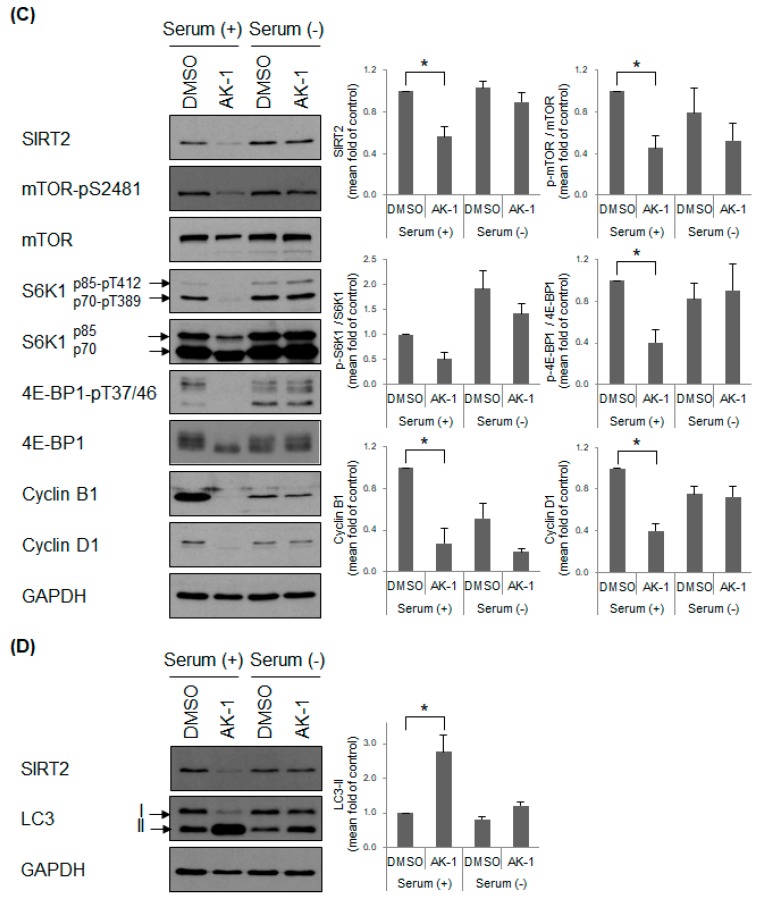
SIRT2-regulated cilia formation and mTOR signaling is dependent on serum. (**A**–**D**) hTERT-RPE1 cells were transfected with control siRNA (siCon) or SIRT2-targeting siRNA (siSIRT2), or treated with 0.1% DMSO or 10 μM AK-1 in the presence or absence of serum for 48 h. (**A**,**B**) The number of ciliated cells (out of 200 cells) was counted and the result was expressed as the mean ± SEM (*n* = 5). * *p* < 0.05; significantly different from siCon-transfected or DMSO-treated cells in the presence of serum (one-way ANOVA followed by Tukey’s HSD test). (**A**–**D**) Expression of SIRT2, mTOR, its substrates, cyclins, and LC3 was determined by Western blotting. Relative expression is presented as the mean ± SEM (*n* = 6). * *p* < 0.05 and # *p* < 0.05; significantly different from control cells in the presence and absence of serum, respectively (Kruskal–Wallis test followed by Dunn’s multiple comparison).

To control cell proliferation, mTOR signaling is sensitive to stimuli such as growth factors. Therefore, we investigated whether SIRT2-dependent cilia formation is dependent on nutrient status. Cells were starved of serum containing growth factors, which resulted in arrest at G0/G1 phase and promoted cilia formation. However, unlike under serum-present conditions, SIRT2 suppression using SIRT2 siRNA and AK-1 did not increase cilia formation above that in control cells under serum-deprived conditions (Figure 4A,B and Figure S2A,B). This suggests that SIRT2 affects primary cilia formation in a proliferative signal-sensitive manner. Furthermore, we examined the effect of SIRT2 on mTOR signaling under serum-depleted conditions. Expression of mTOR-pS2481, p70S6K1-pT389/p85S6K1-pT412, and 4E-BP1-pT37/46 was not affected markedly by AK-1 treatment in the absence of serum (Figure 4C). In addition, the level of cyclin D1, one of the mTOR-regulated proteins, was not changed markedly by AK-1 treatment although the level of cyclin B1 decreased, but not significantly (Figure 4C). AK-1 increased the level of LC3-II, in the presence of serum, but not in the absence of serum (Figure 4D). Overall, it suggests that SIRT2 does not affect mTOR signaling under serum-deprived conditions. Interestingly, serum starvation induced a slight increase in SIRT2 expression although the change was not significant (Figure 4A–C; Lane 1 versus Lane 3 and Lane 2 versus Lane 4). This might be due to a transcriptional upregulation of SIRT2 through a serum responsive element [49]. In addition, the expression of SIRT2 did not significantly decrease following AK-1 treatment in the absence of serum, whereas it fell in the presence of serum (Figure 4B,C; Lane 2 versus Lane 4). It is also probably because serum starvation promotes transcriptional upregulation of SIRT2 as discussed; however, further investigation is needed to examine this in detail. Here, we raised the possibility that the insensitivity of mTOR signaling in AK-1 treated cells under serum-starved conditions might be due to the fact that the expression of SIRT2 was not markedly inhibited by AK-1 treatment. To rule out this possibility, the expression of SIRT2 was abolished using transfection of SIRT2 siRNA and then mTOR signaling was confirmed in the presence and absence of serum. The level of SIRT2 clearly fell in SIRT2 siRNA-transfected cells under both serum-proficient and serum-starved conditions (Figure 4A and Figure S3) although there was still a slight increase in SIRT2 expression under serum-starved conditions (Lane 2 versus Lane 4). mTOR signaling in SIRT2 siRNA-transfected cells did not decrease under serum-deprived conditions, while it fell under serum-proficient conditions (Figure S3). The next question was about the activity of SIRT2 in the presence and absence of serum. AK-1-treated cells showed a higher level of α-tubulin-acetyl K40, a substrate of SIRT2, than control cells in the presence of serum, but not in the absence of serum (Figure S4; Lane 2 versus Lane 4). It suggests that SIRT2 activity becomes insensitive under serum-starved conditions; the upregulation of SIRT2 expression might compensate for the decreased activity of SIRT2 following AK-1 treatment. Unexpectedly, the level of total α-tubulin was downregulated by AK-1 treatment under serum-proficient conditions although the level of other loading controls such as GAPDH and H3 was equal. It needs to be further investigated whether AK-1 exerts a yet-to-be identified function to regulate stability of α-tubulin. Taken together, these data suggest that SIRT2 regulates mTOR signaling following exposure to stimuli such as mitogens and nutrients.

### 2.5. Inhibition of mTOR Induces a Non-Proliferating Status and Increases Primary Cilia Formation

**Figure 5:**
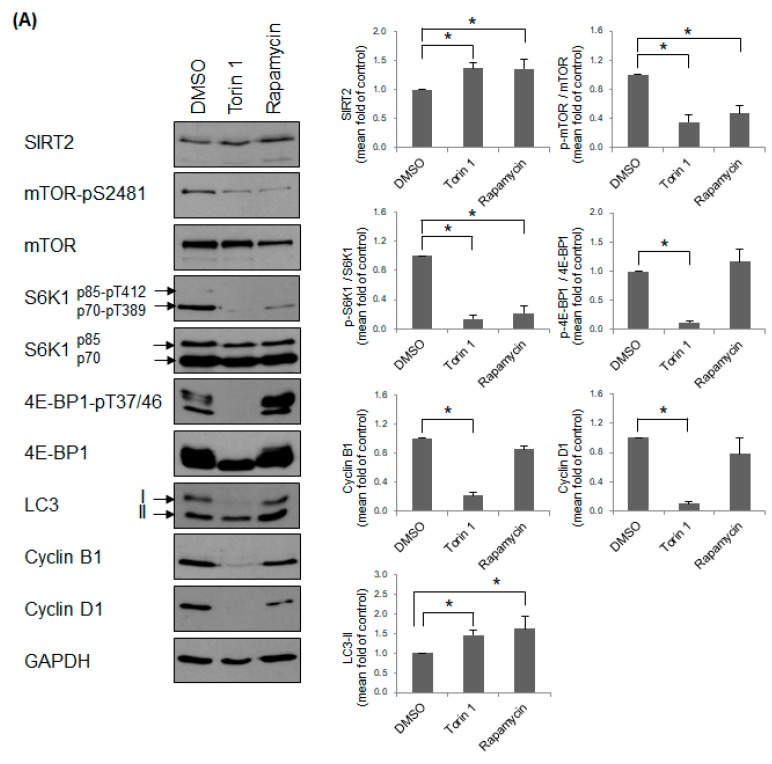

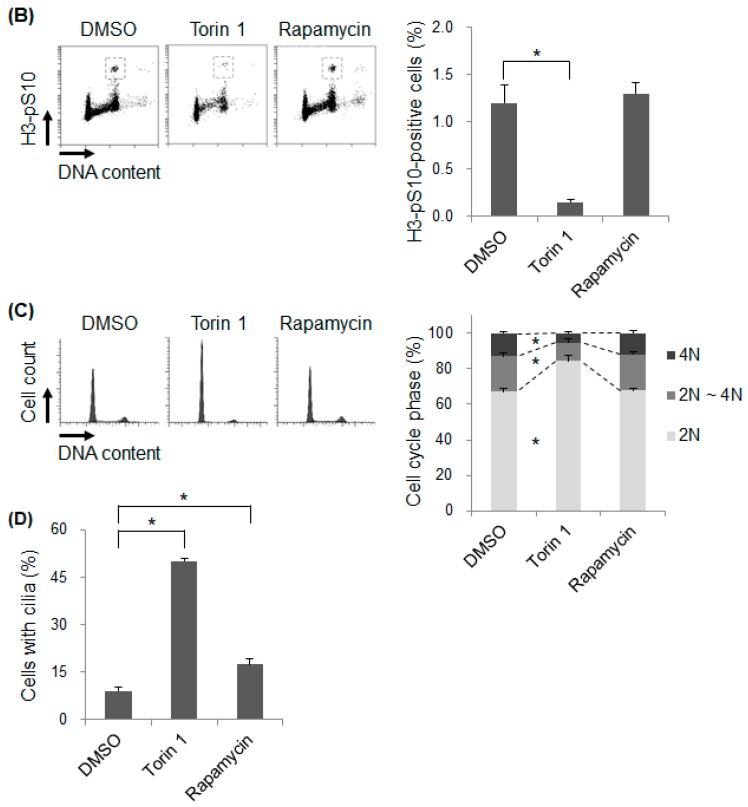
Inhibition of mTOR signaling induces cell cycle arrest and ciliogenesis. (**A**–**D**) hTERT-RPE1 cells were treated with 0.1% DMSO, 0.2 μM torin 1, or 15 μM rapamycin for 48 h. (**A**) Expression of mTOR, its substrates, LC3, and cyclins was determined by Western blotting. Relative expression is presented as the mean ± SEM (*n* = 5). * *p* < 0.05; significantly different from DMSO-treated cells (Kruskal–Wallis test followed by Dunn’s multiple comparison); (**B**,**C**) Mitotic cells and DNA content were determined by flow cytometry after staining cells with an anti-histone H3-pS10 antibody and propidium iodide. The proportion of mitotic cells (**B**) and percentage of cells at each stage of the cell cycle (**C**) are expressed as the mean ± SEM (*n* = 4). * *p* < 0.05; significantly different from DMSO-treated cells (one-way ANOVA followed by Tukey’s HSD test); (**D**) The number of ciliated cells (out of 200 cells) was counted and expressed as the mean ± SEM (*n* = 5). * *p* < 0.05; significantly different from DMSO-treated cells (one-way ANOVA followed by Tukey’s HSD test).

Finally, we investigated whether the effects of mTOR inhibition are similar to those of SIRT2 suppression. ATP-competitive mTOR inhibitors torin 1 and rapamycin inhibit mTORC1/mTORC2 and mTORC1, respectively [50]. mTOR activity was inhibited by treatment of torin 1 and rapamycin in hTERT-RPE1 cells. As expected, the levels of mTOR-pS2481 and p70S6K1-pT389/p85S6K1-pT412 decreased significantly in both torin 1- and rapamycin-treated cells (Figure 5A). In addition, the level of LC3-II increased in both torin 1- and rapamycin-treated cells (Figure 5A). Unexpectedly, the level of SIRT2 also increased in torin 1- and rapamycin-treated cells, suggesting that mTOR acts as a negative regulator of SIRT2 expression. The level of 4E-BP1-pT37/46 in torin 1-treated cells, but not that in rapamycin-treated cells, decreased significantly (Figure 5A); this is because mTORC1-mediated phosphorylation of 4E-BP1 at T37/46 is rapamycin-resistant [51,52,53]. The level of cyclin B1 decreased after treatment with torin 1, but not after treatment with rapamycin (Figure 5A). Consistent with these data, expression of cyclin D1, which is translated in a 4E-BP1-dependent manner [45], did not decrease significantly in rapamycin-treated cells (Figure 5A). This suggests that rapamycin does not inhibit cell cycle progression in hTERT-RPE1 cells. Indeed, we observed a significant decrease in H3-pS10 (Figure 5B), along with the accumulation of a 2N cell population (Figure 5C), only in torin 1-treated cells, but not in rapamycin-treated cells. It indicates that rapamycin does not induce a non-proliferating status in hTERT-RPE1 cells. Finally, treatment with both torin 1 and rapamycin induced significant cilia formation, although induction was more significant in torin 1-treated cells (Figure 5D and Figure S5). Overall, treatment with torin 1, an mTORC1/mTORC2 inhibitor, showed effects similar to those induced by SIRT2-suppression. The next question was whether SIRT2 regulates mTOR signaling via mTORC1 or mTORC2 complex. The activity of mTORC1 is positively regulated by mTORC2 through the phosphorylation of AKT [36,54,55,56]. It raised the possibility that mTORC2 is also involved in the SIRT2-dependent regulation of mTOR signaling. To check the involvement of mTORC2 in SIRT2-suppressed cells, the phosphorylated level of AKT was determined. First, as expected, torin 1 reduced the level of AKT-pS473, while rapamycin did not affect it (Figure S6). In addition, AK-1 decreased the level of AKT-pS473, suggesting that mTORC2 participates in the regulation of mTORC1 in SIRT2-suppressed cells (Figure S4). However, considering that phosphorylation of S6K1 and 4E-BP1, and autophagy inhibition, are dependent on mTORC1 but not on mTORC2, the data suggest that mTORC1 (possibly assisted by mTORC2) signaling mainly regulates cell proliferation and cilia formation.

## 3. Discussion

**Figure 6:**
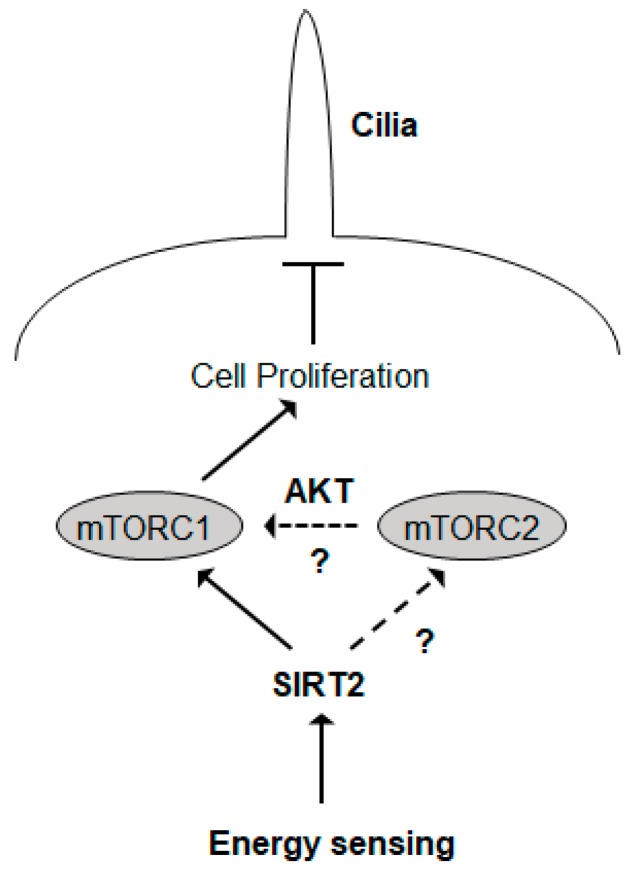
SIRT2 is a critical hub that links nutrient signaling and primary cilia formation. SIRT2 senses growth factor status and controls mTOR signaling. We propose that SIRT2 regulates mainly the mTORC1 complex (SIRT2-mediated regulation of mTORC2 requires further investigation). Activation of mTOR signaling promotes cell proliferation. By contrast, SIRT2 suppression inactivates mTOR signaling and arrests cells at G0/G1 phase. SIRT2 suppression promotes cilia formation after cell cycle arrest. Therefore, SIRT2 plays a role as a link between the energy milieu and ciliogenesis. The significance of black solid arrows was addressed in our study, but that of black dotted arrows needs further investigation.

Here, we show that SIRT2 suppression leads to a cell cycle arrest and subsequent formation of primary cilia by inhibiting mTOR signaling. In addition, we show that the effects of SIRT2 on mTOR signaling and cilia formation were dependent on nutrient status. Overall, the data suggest that SIRT2 is a functional hub that links nutrient signaling and primary cilia formation (Figure 6).

We found that SIRT2 siRNA- and AK-1-treated hTERT-RPE1 cells showed increased primary cilia formation under serum-proficient conditions, but not under serum-starved conditions (Figure 4A,B). This contradicts the report by Zhou et al., which shows that treatment of mouse IMCD3 cells with nicotinamide and siRNA against SIRT2 increases cilia formation and cilia length under starvation conditions [28]. Cilia disassembly upon addition of serum is delayed in SIRT2-deficient cells. While overexpression of wild-type SIRT2 reduces the cilia formation and cilia length, overexpression of an enzymatically inactive SIRT2 mutant-type does not, indicating that SIRT2 deacetylase activity is required for the negative effects on cilia formation [28]. Although the mechanism remains unclear, the different serum-dependent responses reported in the present and previous studies might be due to differences in species and tissue origin of cells (human retinal epithelial cells versus mouse renal epithelial cells) and ciliogenesis pathways (intracellular versus extracellular pathways). Primary cilia are formed via either an intracellular pathway (majority of cells) or an extracellular pathway (polarized epithelial cells) [24,57,58,59]. In hTERT-RPE1 cells, the mother centriole docks to a primary ciliary vesicle in the cytoplasm. Next, the axoneme starts to grow and stretches out to form the ciliary shaft and sheath. The distal part of the cilium is fused with the plasma membrane and projects into the extracellular milieu. By contrast, in mouse IMCD3 cells, the mother centriole docks directly at the apical plasma membrane, and the axoneme initiates growth. These two types of cilia might respond to extracellular stimuli (e.g., serum starvation) to different degrees. However, there is still an unresolved question as to why SIRT2 suppression does not affect cilia formation in hTERT-RPE1 cells used in our study under serum-starved conditions. Unlike other cell lines showing a decrease in mTOR signaling following serum starvation, hTERT-RPE1 cells do not show dramatic reduction of mTOR phosphorylation. Another report also shows that serum starvation does not induce the downregulation of S6K1-pT389, a substrate of mTORC1 in hTERT-RPE1 cells [37]. The insensitivity of mTOR signaling in hTERT-PRE1 cells might produce different results from what mIMCD3 cells did under serum-starved conditions. However, because rapamycin treatment enhances cilia formation in both serum-proficient and serum-starved hTERT-RPE1 cells [37], we think that hTERT-RPE1 cells can functionally sense and relay mTOR signaling under serum-starved conditions.

Expression and activity of SIRT2 upon serum starvation have been studied before. The classic serum responsive element resides within the SIRT2 gene promoter, thereby allowing binding of serum response factor (SRF) protein. Serum deprivation results in upregulation of SIRT2 mRNA in HeLa cells [49]. We also observed that the expression of SIRT2 in hTERT-RPE1 cells was also slightly upregulated by serum deprivation although the change was not significant (Figure 4 and Figure S3; Lane 1 versus Lane 3). By contrast, another report showed no change in the level of SIRT2 protein in U2OS following serum starvation [60]. Interestingly, the level of SIRT2 was upregulated by inhibitors of mTORC1 and mTORC2 (Figure 5A). It suggests that mTOR activity triggers a negative feedback loop affecting SIRT2 expression and SIRT2 communicates with mTOR-dependent energy signaling. Then, the question is whether SIRT2 activity is affected by serum starvation. Following serum deprivation, acetylation of FOXO1 following dissociation from SIRT2 allows binding to ATG7 for autophagy induction [61], indicating that the activity of SIRT2 plays a negative role in autophagy. However, serum starvation of hTERT-RPE1 cells did not affect the level of LC3-II to a marked degree (Figure 4C; Lane 1 versus Lane 3). In addition, the level of acetylated α-tubulin, a substrate of SIRT2, did not change in serum-starved cells (Figure S4; Lane 1 versus Lane 3). Therefore, we suggest that SIRT2 activity is not altered in serum-starved hTERT-RPE1 cells, although this hypothesis requires further study. In other words, ciliogenesis in serum-starved cells might be a SIRT2-independent phenomenon. 

The unresolved question is why does suppression of SIRT2 induce primary cilia formation in the presence of serum? There are several possible explanations. First, our study (Figure 3B) and that of Inoue et al. [8] show basal induction of autophagy in SIRT2-deficient cells. Recent studies propose that inhibiting autophagy or depleting ATG7 reduce both cilium length and number [62]. Therefore, increased basal autophagy in SIRT2-suppressed cells might contribute to cilia formation. However, there is no direct evidence that SIRT2-regulated autophagy increases cilia formation. Second, considering that SIRT2 is a deacetylase for α-tubulin, SIRT2 suppression is expected to promote acetylation of α-tubulin, which is linked to cilia elongation [18]. Indeed, the shortening of cilia by HDAC6-dependent deacetylation was reversed by overexpression of acetyl-mimic (K40Q) α-tubulin [63], indicating that HDAC6 participates in cilia disassembly. Our study also showed that SIRT2 suppression in hTERT-RPE1 cells also enhanced acetylation of α-tubulin and induced cilia elongation. Although the exact mechanism has not been extensively studied, SIRT2 as well as HDAC6 may cooperatively participate in cilia disassembly. Overall, ciliogenesis in serum-proficient cells is a SIRT2-dependent phenomenon. 

Our data suggest that the effect of SIRT2 suppression on primary cilia formation is primarily due to induction of a non-proliferating state. Suppression of SIRT2 expression and activity leads cells to become stationary or quiescent, at least in part via inactivation of mTOR signaling (Figure 2 and Figure 3). The mTOR kinase comprises mTORC1 and mTORC2 complexes [34,64], both of which control cell growth and proliferation. These mTOR signals are affected by nutrient and energy levels; mTORC1 is regulated by growth factors, amino acids, glucose, energy molecules, and stress, whereas mTORC2 is usually unaffected by these stimuli; rather, it is primarily regulated by growth factors [34,64]. While mTORC1 signaling regulates protein synthesis, lipid synthesis, lysosome biogenesis, and autophagy, mTORC2 regulates survival, iron transport, and cytoskeleton reorganization. Although these two signals are quite different with respect to stimuli and function, there is cross-talk between them. mTORC2 activates mTORC1 via AKT phosphorylation [36,54,55,56]. mTORC2 is negatively regulated by mTORC1 through insulin/PI3K signaling, or inhibited directly by mTORC1-dependent S6K1 phosphorylation [34,65]. Due to the complexity of mTOR signaling, we had two hypotheses to explain how SIRT2 suppression inhibits mTORC1 either directly or indirectly through mTORC2. SIRT2 suppression leads to a reduction of AKT-pS473 (Figure S4), suggesting that mTORC2 takes part in the regulation of mTORC1 in SIRT2-suppressed cells. However, mTORC2-independent AKT signaling is also involved in mTORC1 activation [66]. As we lack evidence that SIRT2 suppression directly inhibits mTORC2 activation, it has yet to be determined whether both mTORC1 and mTORC2 act as a regulatory hub in SIRT2-mediated signaling.

If it is indeed the case that the effect of SIRT2 suppression is mediated mainly by mTORC1 signaling, the next question is why treatment with rapamycin, an inhibitor of mTORC1, did not result in cell cycle arrest as in SIRT2-suppressed cells, while torin 1 inhibited mTORC1-dependent functions completely. Studies show that mTORC1 has rapamycin-resistant functions. First, mTORC1-mediated cell proliferation is mediated by S6K1 and 4E-BP1 [67,68], but rapamycin fails to decrease phosphorylation of 4E-BP1 [51,52,53]. This indicates that rapamycin is not sufficient to induce cell cycle arrest. Second, a high dose of rapamycin is required to induce G1 arrest by attenuating phosphorylation of 4E-BP1 and expression of cyclin D1 in breast cancer cell lines [69]. However, we observed that a high dose of rapamycin resulted in the cell death of hTERT-RPE1 cells. In addition, torin 1-medited inhibition of cell proliferation occurred in mouse embryonic fibroblasts, in which rictor, a component of mTORC2, was knocked out, indicating that regulation of cell proliferation by torin 1 is independent of mTORC2 [51]. Our own results show that torin 1, but not rapamycin, reduced phosphorylation of 4E-BP1 and expression of cyclin D1. Taken together, the data presented herein suggest that SIRT2 suppression inhibits mTOR signaling mainly via mTORC1. However, the mechanism by which SIRT2 regulates mTOR signaling to control of the cell cycle and primary cilia formation requires further study. 

Ciliopathy is a group of disorders caused by genetic alterations that affect cilia structure and function. Such conditions include retinal dystrophy (e.g., Leber’s congenital amaurosis), cystic kidney (e.g., polycystic kidney disease and nephronophthisis), mental retardation (e.g., Joubert syndrome), and skeletal malformation (e.g., orofaciodigital syndrome) [70,71]. In addition, primary cilia are thought to paly roles in obesity and cancer [72,73]. Considering that SIRT2 suppression inhibits cell cycle re-entry, SIRT2 may be a therapeutic target for cancer. In addition, SIRT2 may act as a nutrient sensor regulating primary cilia formation, suggesting possible application as a treatment for obesity. 

## 4. Materials and Methods

### 4.1. Cell Culture

Human retinal pigmented epithelial cells immortalized with hTERT (hTERT-RPE1) were obtained from American Type Culture Collection (ATCC, Manassas, VA, USA) and maintained in DMEM:F12 supplemented with 10% fetal bovine serum (FBS), 0.01 mg/mL hygromycin B, 100 U/mL penicillin G sodium, 100 μg/mL streptomycin sulfate, and 0.25 μg/mL amphotericin B. Serum-starved condition was achieved by depleting of FBS for 48 h.

### 4.2. Drug Treatment

AK-1, a SIRT2 inhibitor was purchased from Merck Millipore (Burlington, MA, USA; 566331). mTOR inhibitors, torin 1 (S2827) and rapamycin (S1039) were purchased from Selleckchem (Houston, TX, USA). hTERT-RPE1 cells were treated with AK-1, torin 1 or rapamycin for 48 h.

### 4.3. Small Interfering RNA (siRNA) Transfection

Control and SIRT2 siRNA were synthesized by ST Pharm. Co., Ltd. (Seoul, Korea) and Shanghai GenePharma Co., Ltd. (Shanghai, China), respectively. The siRNA duplexes were as follows: control siRNA sense strand, UGGUUUACAUGUCGACUAAdTdT; SIRT2 siRNA sense strand, CCUAGAGGCCAAGGCUUAAdTdT. hTERT-RPE1 cells were transfected with 20 nM siRNA using Lipofectamine RNAiMax according to the manufacturer’s instructions (Invitrogen, Carlsbad, CA, USA; 13778-150).

### 4.4. Immunofluorescence Staining

hTERT-RPE1 cells were grown on coverslips. The cells were fixed with 3% paraformaldehyde solution at room temperature for 10 min and then permeabilized with 0.5% Triton X-100 at room temperature for 5 min. The cells were incubated with antibody against IFT88 (Proteintech Group Inc., Rosemont, IL, USA; 13967-1-AP) and α–tubulin-acetyl K40 (Sigma-Aldrich, St. Louis, MO, USA; T7451) at 37 °C for 20 min and then incubated with corresponding secondary antibody at 37 °C for 20 min. The nuclei were counterstained with Hoechst 33342. After a final wash with PBS, coverslips were mounted with antifade solution containing para-phenylenediamine and glycerol in PBS. Stained cells were observed under a laser-scanning confocal microscope (Carl Zeiss, Oberkochen, Germany; LSM700). Two hundred cells were randomly selected, and the number of cells containing cilia was counted in a blinded manner. The length of only flat cilia (cilia which are visible in the same plane of focus) was measured to exclude the underestimation of angled cilia (cilia which are not visible in the same plane of focus) (Nikon Instruments Inc., Tokyo, Japan; Ti2E) [74].

### 4.5. Flow Cytometry Analysis

hTERT-RPE1 cells were suspended in PBS, and then, 100% ethanol was added to be the final concentration of 70% ethanol while gently vortexing. The fixed cells were permeabilized with 0.25% Triton X-100 in PBS on ice for 15 min. The cells were incubated with anti-histone H3-pS10 (Merck, Burlington, MA, USA; 06-570) antibody for 2 h, and then incubated with FITC-conjugated goat anti-rabbit IgG (Jackson ImmunoResearch Laboratories Inc., Wester Grove, PA, USA; 111-095-144) at room temperature in the dark for 1 h. Cells were incubated with DNase-free RNase A at 37 °C for 30 min and then with propidium iodide (PI) at 37 °C in the dark for another 30 min. The percentage of cells in each cell cycle phase and H3-pS10-positive cells were determined by flow cytometry.

### 4.6. Western Blotting

hTERT-RPE1 cells were lysed on ice for 10 min using NETN lysis buffer (100 mM NaCl, 1 mM EDTA, 20 mM Tris-HCl, 0.5% Nonidet P-40, 50 mM β-glycerophosphate, 10 mM NaF, and 1 mM Na_3_VO_4_) containing a protease inhibitor cocktail (Merck Millipore, Burlington, MA, USA; 535140). After centrifugation at 12,000× *g* for 5 min, the supernatant was saved as a crude cell extract. This was boiled in Laemmli buffer and loaded onto a SDS-polyacrylamide gel. Western blotting was performed according to a standard protocol. The following antibodies were used for Western blotting: cyclin B1 (sc-752), cyclin D1 (sc-8396), GAPDH (sc-25778), and SIRT2 (sc-20966) were purchased from Santa Cruz Biotechnology, Inc. (Dallas, TX, USA). mTOR-pS2481 (2974), mTOR (2983), p70S6K1-pT389/p85S6K1-pT412 (9205, 9234), p70/p85 S6K1 (2708), 4E-BP1-pT37/46 (2855), and 4E-BP1 (9644) were obtained from Cell Signaling Technology, Inc. (Danvers, MA, USA). LC3 (PM036) was purchased from MBL international (Woburn, MA, USA). Expression levels of each protein were normalized against GAPDH and quantified using ImageJ software.

### 4.7. Data and Statistical Analysis

All assays were repeated more than four times (n ≥ 4). ‘n’ refers to independent values, not replicates. Data are expressed as the mean ± standard error of mean (SEM). Statistical analysis was performed using SPSS software (version 25; IBM, Armonk, NY, USA). The differences in data of the number of cilia-containing cells and each cell cycle phase were subjected to parametric statistical analysis. Differences between two groups were evaluated using an unpaired Student’s *t*-test. Differences between three groups were evaluated using one-way analysis of variance (ANOVA), followed by Tukey’s honest significant difference. Protein expression levels were normalized to that of GAPDH, and expression in SIRT2 siRNA- or drug-treated cells is presented as a fold-change over that observed in control siRNA- or DMSO-treated control cells (whose expression was set to 1). Protein expression was presented after normalization using a method that generates control values with no variance (SEM = 0); this was done to reduce the effects of different blotting exposure times. Such data were subjected to non-parametric statistical analysis. Differences between two groups were evaluated using the Mann–Whitney *U*-test. Differences between three groups were evaluated using the Kruskal–Wallis test followed by Dunn’s multiple comparison. Post-hoc tests were run only if F achieved *p* < 0.05 and there was no significant inhomogeneity. Statistical differences were considered significant at *p* < 0.05 and are indicated by * or #. 

## Supplementary Materials

Supplementary materials can be found at https://www.mdpi.com/1422-0067/21/6/2240/s1.

## Abbreviations

mTOR	Mammalian target of rapamycin
S6K1	Ribosomal protein S6 kinase 1
4E-BP1	Eukaryotic initiation factor 4E binding protein 1
