# Supplementary material for: SIRT2 Affects Primary Cilia Formation by Regulating mTOR Signaling in Retinal Pigmented Epithelial Cells
Source: Int J Mol Sci. 2020 Mar 24;21(6):2240. doi: 10.3390/ijms21062240 (PMC7139600; doi:10.3390/ijms21062240)
Supplement: Supplementary file 1 [file ijms-21-02240-s001.pdf]

Supplementary Materials and Methods

Drug treatment

Chloroquine (C6628) and bafilomycin A1 (B1793) were obtained from Sigma-Aldrich (St. Louis, MO, USA). hTERT-RPE1 cells were treated with chloroquine and bafilomycin A1 for 12 h prior to lysis.

Western blotting

Western blotting was performed according to a standard protocol. The following antibodies were used for Western blotting. α–tubulin-acetyl K40 (T7451) and α–tubulin (PA5-29444) was purchased from Sigma-Aldrich (St. Louis, MO, USA) and Invitrogen (Carlsbad, CA, USA; 13778-150), respectively. AKT (4691) and AKT-pS473 (4060) was purchased from Cell Signaling Technology, Inc. (Danvers, MA, USA). H3 (07-690) was obtained from Merck Millipore (Burlington, MA, USA AKT)

Supplementary Figures

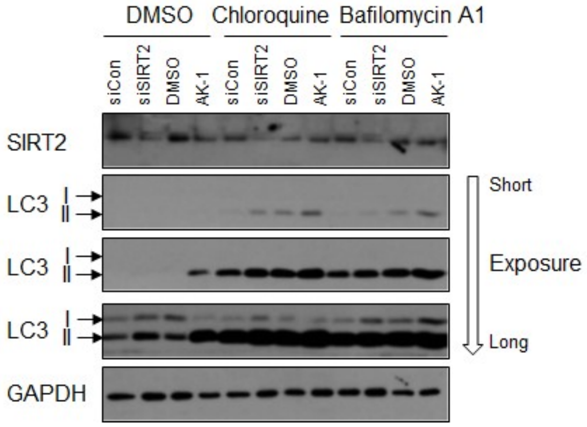

Figure S1. SIRT2 is a negative regulator of autophagy induction. hTERT-RPE1 cells were transfected with control siRNA (siCon) or SIRT2-targeting siRNA (siSIRT2), or treated with 0.1% DMSO or 10 μM AK-1, a SIRT2-specific inhibitor, for 48 h. The autophagic clearance was blocked by treating cells with 100 μM chloroquine or 10 nM bafilomycin A1 for 12 h prior to lysis.

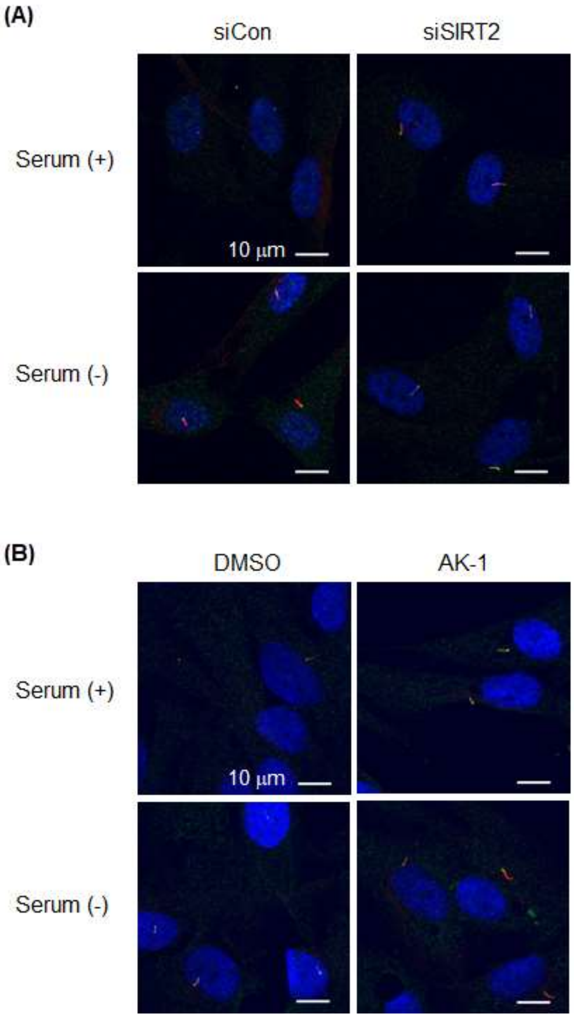

Figure S2. Suppression of SIRT2 increases ciliogenesis in the presence of serum, but not in the absence of serum. hTERT-RPE1 cells were transfected with control siRNA (siCon) or SIRT2-targeting siRNA (siSIRT2) (**A**), or treated with 0.1% DMSO or 10 μM AK-1, a SIRT2-specific inhibitor (**B**), in the presence or absence of serum for 48 h. Cilia were visualized by staining with antibodies specific for IFT88 (green) and α-tubulin-acetyl K40 (red), and nuclei were stained with Hoechst. Scale bars, 10 μm.

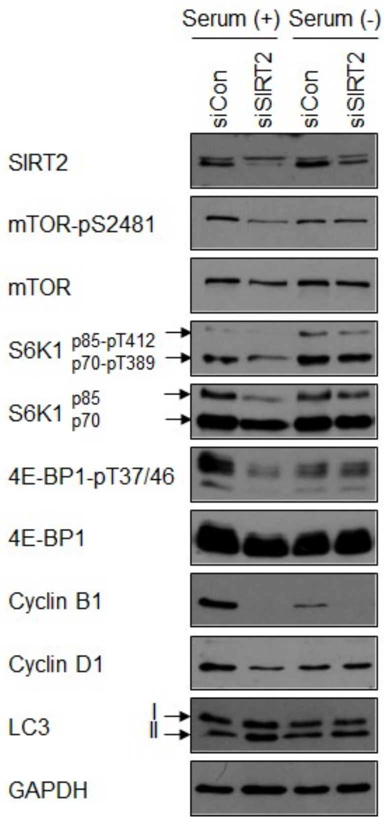

Figure S3. The SIRT2-regulated mTOR signaling is dependent on serum presence. hTERT-RPE1 cells were transfected with control siRNA (siCon) or SIRT2-targeting siRNA (siSIRT2) in the presence or absence of serum for 48 h. Expression of SIRT2, mTOR, its substrates, cyclins and LC3 was determined by western blotting.

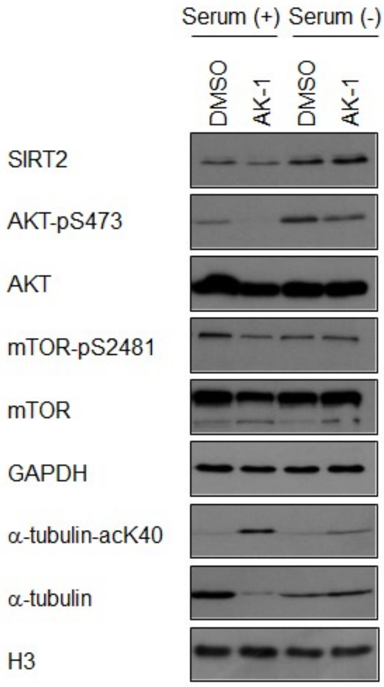

Figure S4. The activity of SIRT2 and AKT is inhibited in AK-1-treated cells under serum-proficient conditions. hTERT-RPE1 cells were treated with 0.1% DMSO or 10 μM AK-1 in the presence or absence of serum for 48 h. Expression of SIRT2, α-tubulin-acetyl K40 and, AKT-pS473 was determined by western blotting.

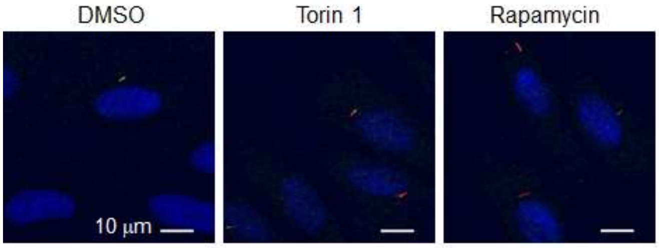

Figure S5. Suppression of mTOR signaling increases ciliogenesis. hTERT-RPE1 cells were treated with 0.1% DMSO, 0.2 μM torin 1, or 15 μM rapamycin for 48 h. Cilia were visualized by staining with antibodies specific for IFT88 (green) and α-tubulin-acetyl K40 (red), and nuclei were stained with Hoechst. Scale bars, 10 μm.

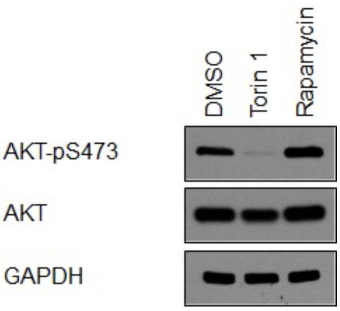

Figure S6. Inhibition of mTORC2, not mTORC1, results in inactivation of AKT. hTERT-RPE1 cells were treated with 0.1% DMSO, 0.2 μM torin 1, or 15 μM rapamycin for 48 h. Expression of AKT phosphorylation at S473 was determined by western blotting.
